# EGFR-IL-6 Signaling Axis Mediated the Inhibitory Effect of Methylseleninic Acid on Esophageal Squamous Cell Carcinoma

**DOI:** 10.3389/fphar.2021.719785

**Published:** 2021-07-30

**Authors:** Yu Wang, Xianghe Liu, Guanghui Hu, Chenfei Hu, Yang Gao, Miaomiao Huo, Hongxia Zhu, Mei Liu, Ningzhi Xu

**Affiliations:** Laboratory of Cell and Molecular Biology and State Key Laboratory of Molecular Oncology, National Cancer Center/National Clinical Research Center for Cancer/Cancer Hospital, Chinese Academy of Medical Sciences and Peking Union Medical College, Beijing, China

**Keywords:** MSA, ESCC, EGFR, miR-146a, IL-6

## Abstract

Epidemiological and experimental evidence indicate that selenium is associated with a reduced risk of some cancers, including esophageal cancer. However, the exact mechanism is still unclear. In the present study, we used esophageal squamous cell carcinoma (ESCC) cell lines and animal models to explore the anti-cancer mechanism of methylseleninic acid (MSA). Firstly, MSA treatment dramatically attenuated Epidermal Growth Factor Receptor (EGFR) protein expression but did not alter mRNA levels in ESCC cells. On the contrary, EGFR overexpression partly abolished the inhibitory effect of MSA. With a microRNA-array, we found MSA up-regulated miR-146a which directly targeted EGFR, whereas miR-146a inhibitor antagonized MSA-induced decrease of EGFR protein. We further used 4-nitroquinoline-1-oxide (4NQO)-induced esophageal tumor mice model to evaluate the inhibitory effect of MSA *in vivo*. MSA treatment significantly decreased the tumor burden and EGFR protein expression in tumor specimens. Furthermore, MSA treatment inhibited EGFR pathway and subsequntly reduced Interleukin-6 (IL-6) secretion in the supernatant of cancer cell lines. MSA-induced IL-6 suppression was EGFR-dependent. To further evaluate the association of IL-6 and the anti-tumor effect of MSA on esophageal cancer, we established the 4NQO-induced esophageal tumor model in IL-6 knock-out (IL-6 KO) mice. The results showed that IL-6 deficiency did not affect esophageal tumorigenesis in mice, but the inhibitory effect of MSA was abolished in IL-6 KO mice. In conclusion, our study demonstrated that MSA upregulated miR-146a which directly targeted EGFR, and inhibited EGFR protein expression and pathway activity, subsequently decreased IL-6 secretion. The inhibitory effect of MSA on esophageal cancer was IL-6 dependent. These results suggested that MSA may serve as a potential drug treating esophageal cancer.

## Introductions

Selenium (Se), an essential and unique trace element, plays a key role in human health and disease. Schwartz and Foltz found selenium prevented liver necrosis in rats in 1950s (Schwarz and Foltz, 1958), thereafter, benefits of selenium in human health have been rapidly recognized. Epidemiologic studies suggested that serum selenium levels inversely correlated with cancer risk (Willett et al., 1983). A significantly inverse correlation was reported between serum selenium levels and the incidence of esophageal squamous cell carcinoma (ESCC) in Linxian, China, an area with high incidence of esophageal cancer (Mark et al., 2000). Furthermore, selenomethionine possessed a protective effect among subjects with mild esophageal squamous dysplasia at baseline (Limburg et al., 2005).

Several selenium compounds have been shown to selectively target tumour cells (Ghose et al., 2001; Nilsonne et al., 2006), thus making selenium promising to be an antitumour drug. Chemical forms and doses are key factors affected selenium effectiveness as anti-cancer agents. Among these forms, Monomethylselenol is a highly reactive species and probabaly the most efficient compound to induce apoptosis in malignant cells. Methylseleninic acid (MSA) is a synthetic selenium compound acting as an immediate precursor of methylselenol (Ip et al., 2000; Wallenberg et al., 2014). The anticancer effects of MSA have been evident in human lung (Poerschke et al., 2008), prostate (Jiang et al., 2001; Jiang et al., 2002; Lee et al., 2006; Li et al., 2007), breast cancer (Jariwalla et al., 2009). Moreover, MSA considerably reduced tumor growth in prostate and colon cancer xenograft models, but did not induce animal weight loss or other signs of systemic toxicity (Lee et al., 2006; Wang et al., 2009; Zeng and Wu, 2015). However, the exact mechanism of MSA in the prevention of esophageal carcinogenesis is still unclear.

Epidermal growth factor receptor (EGFR), a tyrosine kinases receptor (RTK) of ErbB family, is over-expressed in some cancers (Salomon et al., 1995; Mendelsohn and Baselga, 2000), including esophageal squamous cell carcinoma (ESCC) (Hanawa et al., 2006; Wang et al., 2013; Shang et al., 2014). High EGFR expression was associated with invasion, metastasis and poor prognosis (Wang et al., 2013). EGFR activation stimulates several pathways as RAS/RAF/MEK/ERK, PI3K/AKT, Src kinases, and STAT signaling (Lemmon and Schlessinger, 2010). Interleukin-6 (IL-6) is a key inflammatory cytokine participating in inflammation-associated carcinogenesis. IL-6 is also a target of STAT3, which serves as an important component of EGFR downstream signaling (Schafer and Brugge, 2007; Heikkilä et al., 2008). IL-6 was reported to positively associate with angiogenesis, EMT, and poor prognosis in esophageal cancer (Chen et al., 2013; Chen et al., 2014). Rooprai *et al* reported that selenium downregulated EGFR mRNA levels in human biopsy-derived glima cells (Rooprai et al., 2007). Seleno-L-methionine (SeMet) reduced EGFR transcription and protein stability in human lung cancer cell lines (Shin et al., 2007). These two studies provided clues that selenium may affect EGFR expression, but the mechanism is unclear. Recent studies implied that inhibiting EGFR pathway in cancer cells modulated cytokine secretion (Zhang et al., 2016; Suh et al., 2017). Our previous studies have demonstrated that MSA inhibited cell growth and induced apoptosis in ESCC cells by attenuating β-catenin/TCF pathway and modulating HDAC activity (Zhang et al., 2010; Hu et al., 2015). Whether EGFR was involved in the inhibitory effect of MSA have not been studied.

In the present study, we firstly verified that MSA decreased the incidence of esophageal tumor formation in 4NQO-induced mice model. We further the mechanism and found that MSA downregulated EGFR by inducing miR-146a and subsequently inhibited activation of EGFR pathway. MSA decreased the secretion of IL-6. These results indicated that the inhibitory effect of MSA in ESCC was IL-6 dependent.

## Materials and Methods

### Cell Culture and Transfections

The KYSE series was kindly provided by Dr. Yutaka Shimada (Kyoto University, Kyoto, Japan). KYSE150, KYSE510 were cultured in RPMI1640 (Bioroc™, China) containing 10% fetal bovine serum (FBS) and supplemented with 1% penicillin/streptomycin at 37°C with 5% CO_2_. Lipofectamine 3000 transfection reagent (Invitrogen, Carlsbad, CA, United States) was used for transfection according to the protocol provided by the manufacturer. Antagomir-146a was synthesized from Ribo Biotechnology (Guangzhou, China). pcDNA6-EGFR plasmid was provided by Mien-Chie Hung (Addgene plasmid # 42665) (Hsu and Hung, 2007). EGFR small interfering RNA (siRNA) (NM_005228, sequence: 5’AGC​UAU​GAG​AUG​GAG​GAA​GAC​GGC​G3’) was purchased from Integrated DNA Technologies (IDT, Coralville, IA, United States).

### Cell Viability Analysis

Cells were seeded into 96-well plate by 2×10^3^ per well, incubated overnight, and then treated with MSA (541281, Sigma) at indicated concentrations. Cell viability was assessed every 24 h for 3 days using the CCK8 assay (Dojindo, Kumamoto, Japan). The detailed procedure was described previously (Pan et al., 2018).

### Colony Formation

KYSE 150 and KYSE 510 cells were seeded into six-well plates by 500 per well, respectively. The experiment was performed as previously decribed (Liu et al., 2018).

### TaqMan Real-Time PCR microRNA Array

The differentially expressed miRNAs in KYSE 150 cells treated with or without MSA were identified by TaqMan Real-time PCR microRNA Array A (V2.0) (Applied Biosystems, CA). All reactions were performed according to the manufactures’ protocol as described previously (Wang et al., 2015). The data was analyzed by using the SDS 2.0.1 software (automatic baseline, threshold 0.2) and Data Assist v2.0 software (Applied Biosystems, CA). U6 was used as endogenous control for miRNA expression analysis.

### Quantitative Real-Time PCR

The expression of EGFR, IL-6 mRNAs was measured by using qRT-PCR. Total RNA extraction and reverse transcription were performed as described (Yang et al., 2020). qPCR was performed with SYBR Green PCR reagents (Applied Biosystems). GAPDH was used as the control gene. The primers used were as follows: GAPDH, 5’GCT​CCT​CCT​GTT​CGA​CAG​TCA3’/5’ACC​TTC​CCC​ATG​GTG​TCT​GA3’; EGFR, 5’AGG​CAC​GAG​TAA​CAA​GCT​CAC3’/5’ATG​AGG​ACA​TAA​CCA​GCC​ACC3’; IL-6, 5’ACT​CAC​CTC​TTC​AGA​ACG​AAT​TG3’/5’ CCA​TCT​TTG​GAA​GGT​TCA​GGT​TG3’.

For miRNA analysis, reverse transcription and stem-loop real-time RT-PCR were performed as described (Chen et al., 2005). For normalization, U6 was used as endogenous control.

All the quantitative real-time PCR of each sample was performed in triplicate on StepOnePlus™ Real-Time PCR System (Applied Biosystems, Carlsbad, CA, United States) and analyzed with StepOne Software.

### Western Blot

Cells were harvested and washed in PBS and lysed in RIPA buffer (9806, Cell Signaling Technology). Western blot analysis was performed by using the conventional protocols as described previously (Ma et al., 2016). Primary antibodies were used including EGFR (sc-03, Santa Cruz Biotechnology), phospho-EGFR (Tyr1068) (3777, Cell Signaling Technology), phospho-Stat3 (Tyr705) (9145, Cell Signaling Technology), Stat3 (9132, Cell Signaling Technology), phospho-Akt (Ser473) (9271, Cell Signaling Technology), Akt (9272, Cell Signaling Technology), GAPDH (60004-1-Ig, Proteintech). After extensively washed, the membranes were then incubated with secondary antibodies (Zhongshan Golden Bridge Biotechnology Company, Beijing, China) for 1 h at room temperature. Signals were detected using enhanced chemiluminescence (Engreen, Beijing, China). The bands intensities were analyzed using ImageJ software.

### Animal Experiments

IL-6^tm1Kopf^ mice, which are IL-6 gene-knockout (IL-6 KO) mice, were purchased from Model Animal Research Center of Nanjing University. 6-week-old C57BL/6 J mice and IL-6 KO mice were used for establishing the 4-nitroquinoline 1-oxide (4NQO)-induced esophageal tumor model. The carcinogen 4NQO (N8141-5G, Sigma) stock solution was prepared in propylene glycol at 5 mg/ml and stored at 4°C and added to the drinking water at a concentration of 100 μg/ml (Tang et al., 2004). After 12 weeks of the carcinogen treatment, mice were randomly divided into the experimental group and the control group and received drinking water with or without MSA, respectively. The MSA stock solution was prepared in ddH_2_O at 0.04 g/ml and added to the drinking water for the mice at the concentration of 0.02 mg/ml. With MSA treatment or not for 12 weeks, mice were sacrificed and whole esophagi were opened longitudinally, and tumors were counted and tumor volumes were calculated according to the formula Length×Width^2^×0.5. Tissues were fixed overnight in 4% paraformaldehyde, paraffinembedded and sectioned. All animal experiments were approved by the Institutional Animal Care and Use Committee (IACUC) of the Cancer Hospital, Chinese Academy of Medical Science.

### Cytokine Antibody Array and ELISA Analysis

KYSE150 and KYSE510 were treated with MSA (2 and 5 μM, respectively) for 24 h, then cells were digested and reseeded in 100 mm dish (1.2 × 10^6^ cells/dish) with the same volume medium without fetal bovine serum, 24 h later, collected the conditioned media for the cytokine antibody array. Cytokine antibody array (Raybiotech) was performed according to manufacturer’s protocol. The complete array maps (AAH-CYT-G5 Array 5) can be found at https://www.raybiotech.com/human-cytokine-array-g5-4/.

According to the manufacturer’s protocols, human IL-6 levels in the supernatant and mouse IL-6 levels in the serum were measured by ELISA Kits (#D6050 and #M6000B, R&D Systems), respectively.

### Immunohistochemistry

Sections of 5 μm thickness were deparaffinized in xylene and rehydrated in graded ethanol. Antigen retrieval was carried out in 10 mM sodium citrate buffer (pH 6.0). The endogenous peroxidase activity was quenched by 3% hydrogen peroxide for 20 min, and then the slides was blocked by 5% BSA for 1 h to avoid nonspecific staining. The subsequent immunostaining were performed as described previously (Wang et al., 2016). The primary antibody against EGFR (sc-03-G, 1:1,000, Santa Cruz Biotechnology), PCNA (sc-7907, 1:1,000, Santa Cruz Biotechnology) CD31 (77699, 1:1,000, Santa Cruz Biotechnology) was used. We also performed histological analyses by using H&E staining.

### Statistical Analysis

Data with error bars were shown as mean ± SD. The two-tailed Student’s *t-*test was performed to analyze the significance of differences between treatment and control group. *p* < 0.05 was designated statistically significant. GraphPad Prism software was used to do the calculations.

## Reaults

### EGFR Mediated the Inhibitory Effect of MSA in ESCC Cells

**FIGURE 1:**
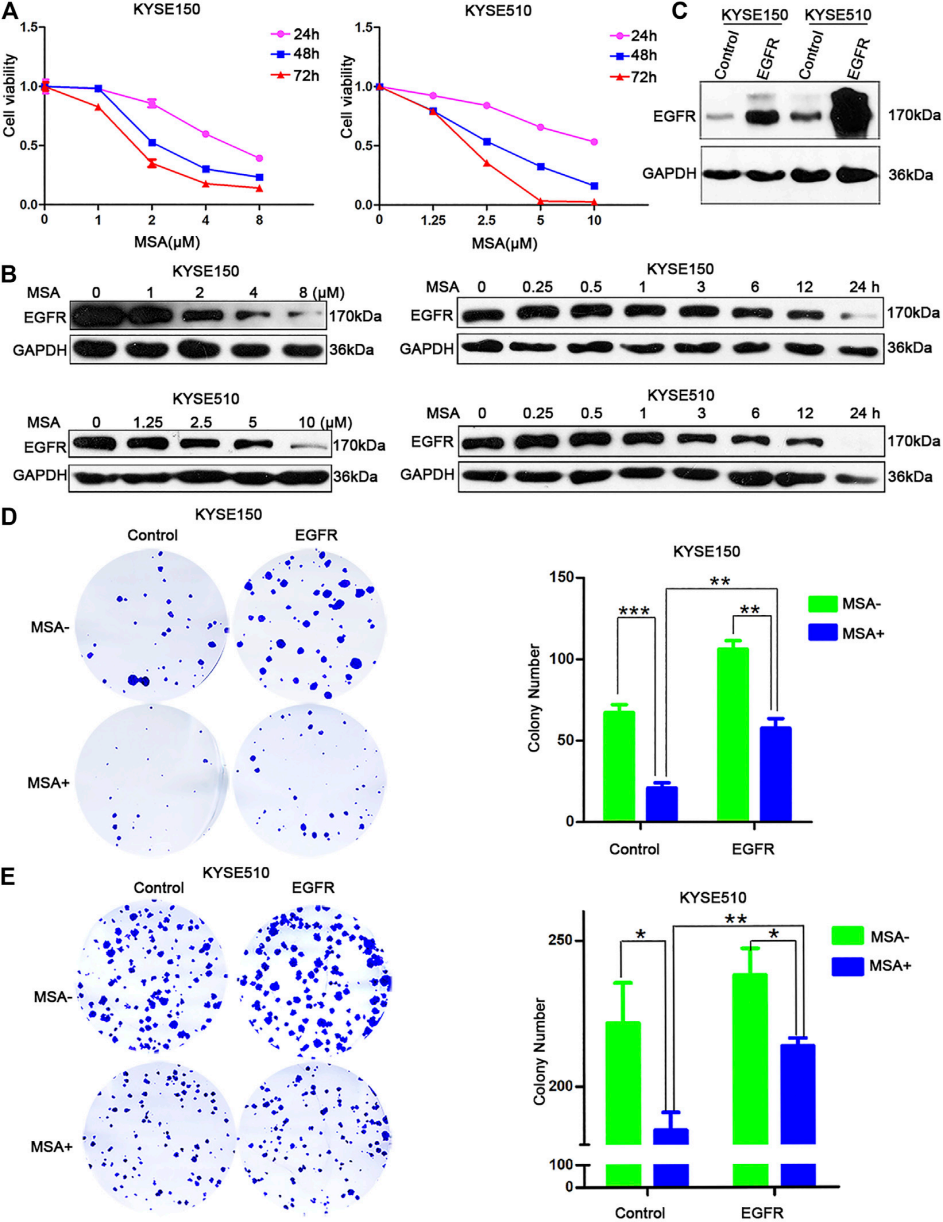
EGFR mediated the inhibitory effect of MSA in ESCC cells. **(A)** Growth inhibition of MSA were measured by CCK8 assay in KYSE150 and KYSE510 cells. Cells were treated with the indicated concentration of MSA for 24, 48 and 72 h, respectively. Data are represented as mean ± SD, *n* = 3. **(B)** KYSE150 and KYSE510 cells were treated with MSA at different concentrations for 24 h (left), or treated with a fixed concentration (2 μM for KYSE150, 5 μM MSA for KYSE510) at different time points (right) and harvested. EGFR protein levels were evaluated by western blotting. GAPDH was used as internal control. **(C)** KYSE150 and KYSE510 cells were transfected with EGFR expression plasmid or the empty control plasmid, and then treated with or without MSA for 24 h, respectively. EGFR protein levels were evaluated by western blotting. GAPDH was used as internal control. **(D,E)** Colony formation assay of KYSE150 **(D)** and KYSE510 **(E)** transfected with EGFR expression plasmid or the empty control plasmid, 12 h afer transfection, the cells were digested, reseeded and treated with or without MSA (1 μM for KYSE150, 2.5 μM for KYSE510) for 9 days, respectively. Results are shown as mean ± SD, *n* = 3.

We found MSA significantly supressed the growth of KYSE150 and KYSE510 cells (Figure 1A), consistant with our previous reports (Zhang et al., 2010; Hu et al., 2015). To better understand the molecular mechanisms by which MSA inhibits tumor cell growth, we performed RNA sequencing (RNA-seq) on KYSE150 and KYSE510 cells treated with or without MSA, respectively. The results revealed 465 commonly differentially expressed genes (Fold change ≥ 2, false discovery rate (FDR) < 0.001) (Supplementary Figure S1A). KEGG pathway analysis reported a number of enriched terms, such as PI3K-Akt signaling pathway, MAPK signaling pathway and Ras signaling pathway (Supplementary Figure S1B). Results indicated that MSA may affect the EGFR pathway. To determine whether EGFR could be supressed by MSA treatment, Western blotting were performed in KYSE150 and KYSE510 cells treated with or without MSA, respectively. Interestingly, we found MSA reduced EGFR protein level in a dose- and time-dependent manner (Figure 1B).

Furthermore, to examine whether MSA suppressed cell growth by downregulating EGFR in ESCC cells, KYSE150 and KYSE510 cells were transfected with EGFR expression plasmid or the empty control plasmid (Figure 1C), and then treated with or without MSA, respectively. The results revealed that EGFR overexpression could promote colony formation of ESCC cells and partly abolish the inhibitory effect of MSA (Figures 1D,E).

### MSA Down-Regulated EGFR Via Up-Regulating miR-146a

**FIGURE 2:**
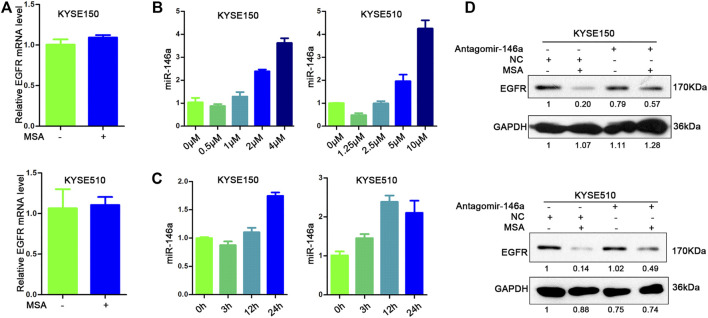
MSA down-regulated EGFR via up-regulating miR-146a. **(A)** KYSE150 and KYSE510 cells were treated with MSA for 24 h (2 and 5 μM, respectively); EGFR mRNA was determined by quantitative real-time PCR. Values are normalized to the untreated cells. **(B,C)** miR-146a expression was detected by TaqMan Real-time PCR in KYSE150 and KYSE510 cells treated with MSA at different concentration for 24 h **(B)** or treated with a fixed concentration (2 μM for KYSE150, 5 μM for KYSE510) at different time points as indicated. **(C)**. U6 was used as the endogenous control. Values are normalized to the corresponding untreated cells, respectively. Bars represent the mean ± SD (*n* = 3) for each treatment. **(D)** KYSE150 and KYSE510 cells transfected with a negative control (NC) or antagomir-146a with or without MSA treatment for 24 h (2 μM for KYSE150 and 5 μM for KYSE510) were harvested and EGFR levels were determined by western blotting. GAPDH was used as internal control.

As mentioned above, MSA treatment could downregulate EGFR protein, but didn’t influence the mRNA level of EGFR in both KYSE150 and KYSE510 cells (Figure 2A). Next, we compared the expression of microRNAs in KYESE150 cells treated with or without MSA by TaqMan Real-time PCR microRNA array. The Analysis of the array data showed that 21 microRNAs changed more than 2-fold in expression between the two samples (Supplementary Table 1). Among these 21 miRNAs, we found miR-146a could directly target EGFR, which have been reported (Li et al., 2010; Kogo et al., 2011). So we chose miR-146a to further detected. As showed in Figures 2B,C, miR-146a showed a dose- and time-dependent increase with MSA treatment. And, antagomir-146a could attenuate MSA-induced EGFR down-regulation (Figure 2D). These findings demonstrated that MSA could down-regulate EGFR via inducing miR-146a.

### MSA Inhibited Tumor Growth of Esophageal Cancer *In Vivo*

**FIGURE 3:**
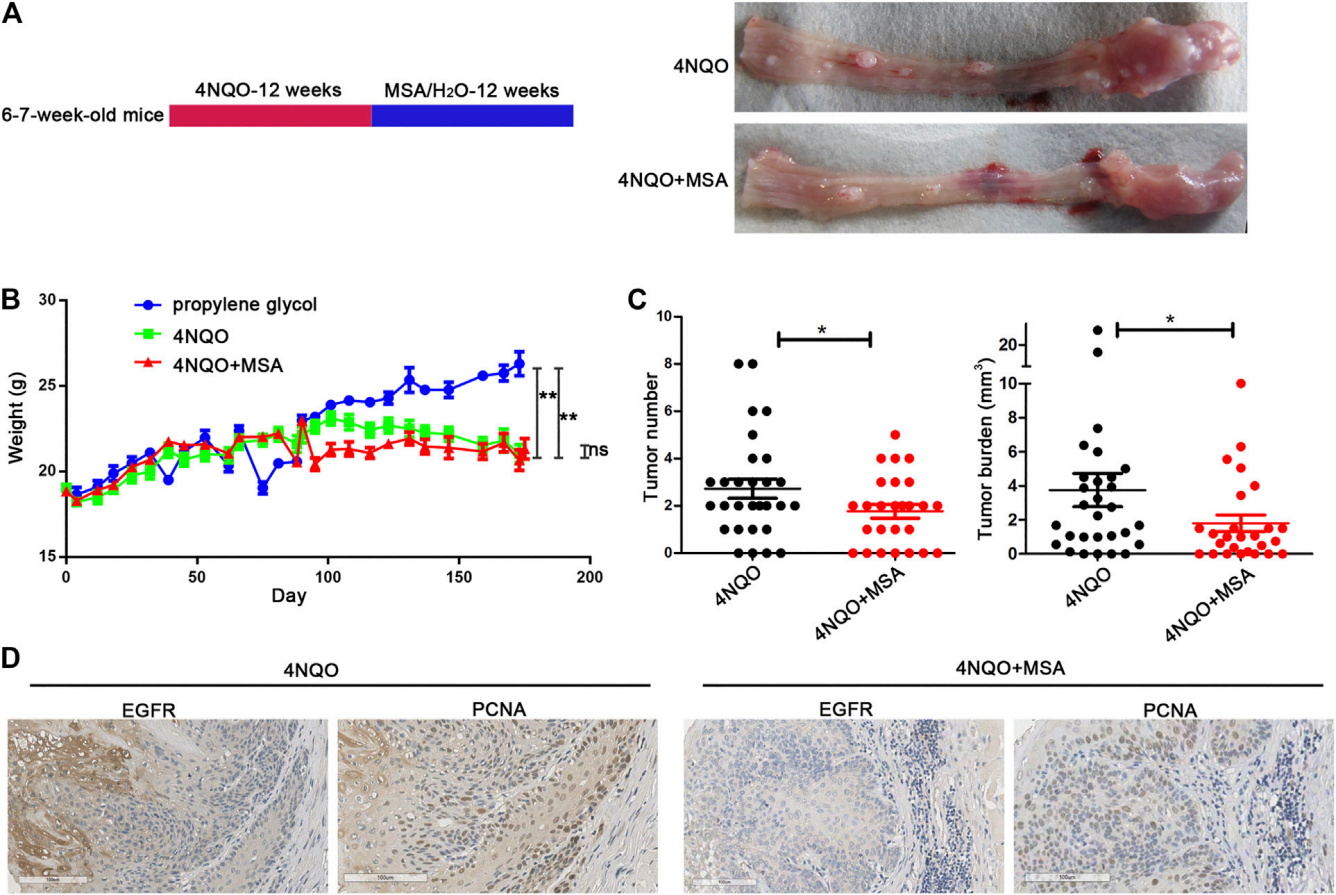
MSA inhibited esophageal tumor growth *in vivo*. **(A)** Schematic representation of 4NQO mice model. 6–7 week-old mice were given drinking water containing 4NQO (100 μg/ml) for 12 weeks. Then mice were randomly divided into two group with treatment of drinking water containing MSA (0.02 mg/ml) or pure water. Twelve weeks later, the mice were sacrificed for esophageal tumor analysis. **(B)** Body weight of mice were measured. Data are expressed as mean ± SD. **p < 0.01; ns, non significant. **(C)** Tumor number and tumor budern in the mice esophagi. C57+4NQO (*n* = 29), C57+4NQO + MSA (n = 26), bars represent the mean ± SD for each group. *p < 0.05. **(D)** Representative photomicrographs of immunostaining of EGFR and PCNA in the mice esophageal tissue.

To explore the anti-tumor activity of MSA *in vivo*, we constructed a 4NQO-induced esophageal tumor mice model using C57 mice (Tang et al., 2004). After 12 weeks of carcinogen exposure, the mice were subjected to MSA treatment for another 12 weeks (Figure 3A). The results showed that the MSA-treated mice had almost the same body weight, less tumors and lower tumor burden compared with the 4NQO group without MSA treatment (Figures 3B,C). To assess whether MSA could decrease the expression of EGFR *in vivo*, we investigated EGFR expression in tumor tissues by IHC staining. As shown in Figure 3D, MSA treatment indeed supressed EGFR expression *in vivo*. We also detected the expression of Proliferating Cell Nuclear Antigen (PCNA). As expected, MSA could attenuate the expression of PCNA in MSA treated mice compared with the control mice.

### MSA Inhibited IL-6 Production

**FIGURE 4:**
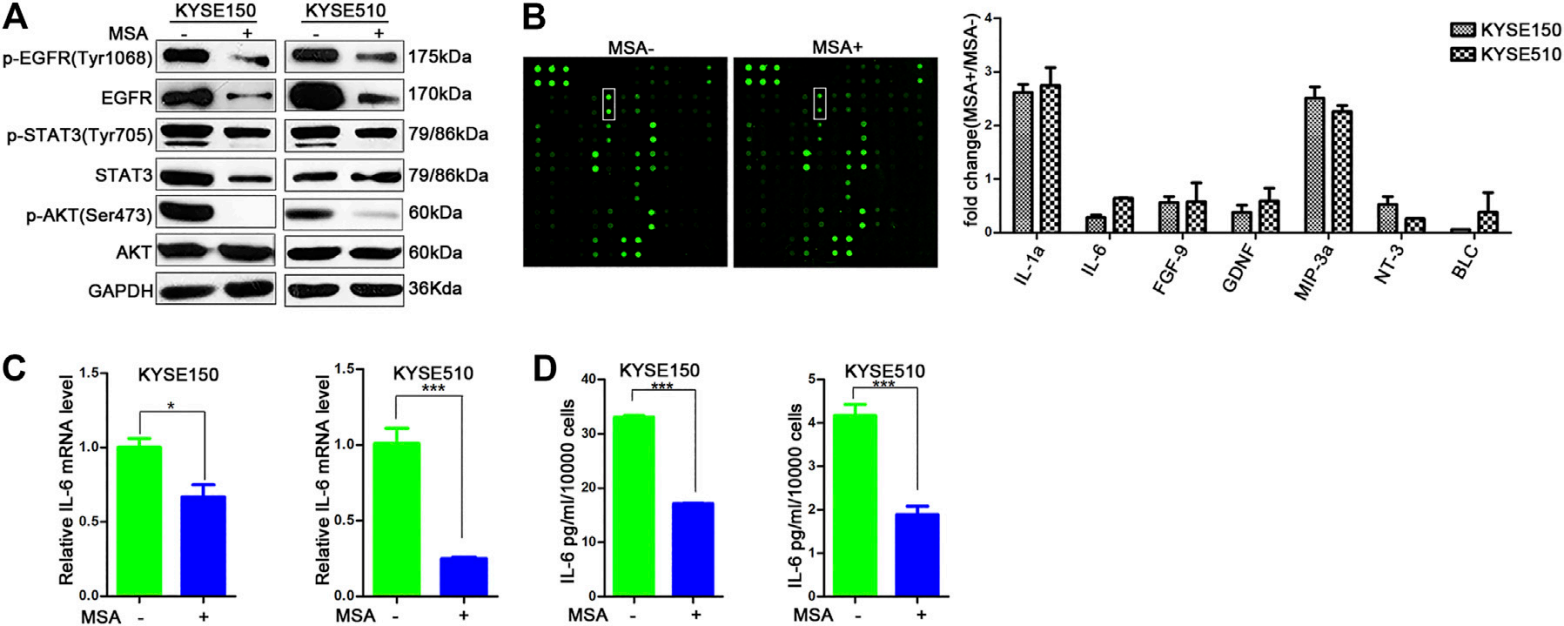
MSA inhibits IL-6 production. **(A)** KYSE150 and KYSE510 cells were treated with MSA for 24 h (2 μM for KYSE150 and 5 μM for KYSE510), then cells were harvested and analyzed by western blotting. GAPDH was used as internal control. **(B)** Conditional media were collected and analyzed by cytokine antibody array. The value represented the average signal density ratio of MSA treatment condition media (MSA+) to the control condition media (MSA-). Mean ± SD (n = 2). **(C)** KYSE150 and KYSE510 cells were treated with or without MSA for 24 h, and the IL-6 mRNA levels were determined by q-RT-PCR. Bars represent the mean ± SD (*n* = 3) for each treatment. **(D)** KYSE150 and KYSE510 cells were treated with MSA for 24 h (2 μM for KYSE150 and 5 μM for KYSE510), then cells were digested and equal count of cells were reseeded with the same volume medium, 24 h later, collected the conditional media and IL-6 levels were determined by ELISA. Bars represent the mean ± SD (*n* = 2) for each treatment. *p < 0.05; ***p < 0.001.

As shown above, MSA could down-regulate EGFR protein. Next, we examined the downstream signaling molecules of EGFR pathway. As Figure 4A showed, the phosphorylation of STAT3, AKT were down-regulated upon MSA treatment. All these indicated that MSA could inhibit the activation of EGFR pathway in ESCC cells. We wonder whether MSA could affect the cytokine secretory. To this end, KYSE150 and KYSE510 cells were treated with MSA or not for 24 h, then the cells were digested and reseeded at the same amount in RPMI1640 without fetal bovine serum, another 24 h later, the supernatant was harvested and tested by the cytokine array. Among the 80 cytokines, 7 cytokines displayed a significant difference with more than 1.5-fold in expression both in the two cell lines treated with or without MSA, respectively. The levels of IL-1a and MIP-3a were higher and IL-6, FGF9, GDNF, NT3, and BLC were lower in the supernatant of MSA-treated cells than that of the untreated cells (Figure 4B). It was already well known that IL-6 regulates immune and inflammatory responses and is involved in tumor progression (Hodge et al., 2005; Iliopoulos et al., 2009). So we firstly chose IL-6 to further study. We evaluated the levels of IL-6 at transcription levels. As shown in Figure 4C, IL-6 mRNA levels were down-regulated in the cells treated with MSA. In accord with the cytokine array, ELISA results also showed a significant decrease of IL-6 in the supernant of ESCC cells with MSA treatment (Figure 4D). Furthermore, we also measured the IL-6 level in the mouse serum by the ELISA Kit (#M6000B, R&D Systems). Although the serum level of IL-6 in 4NQO-induced mice was a little lower than the minimum detectable amount (7.8 pg/ml), it was higher than the levels of IL-6 in the serum of 4NQO-induced mice with MSA treatment and normal C57 mice (data not shown). Even so, our results may provide clues to imply that MSA could inhibit IL-6 production both *in vitro* and *in vivo*.

### EGFR Involved in MSA-Induced IL-6 Downregulation

**FIGURE 5:**
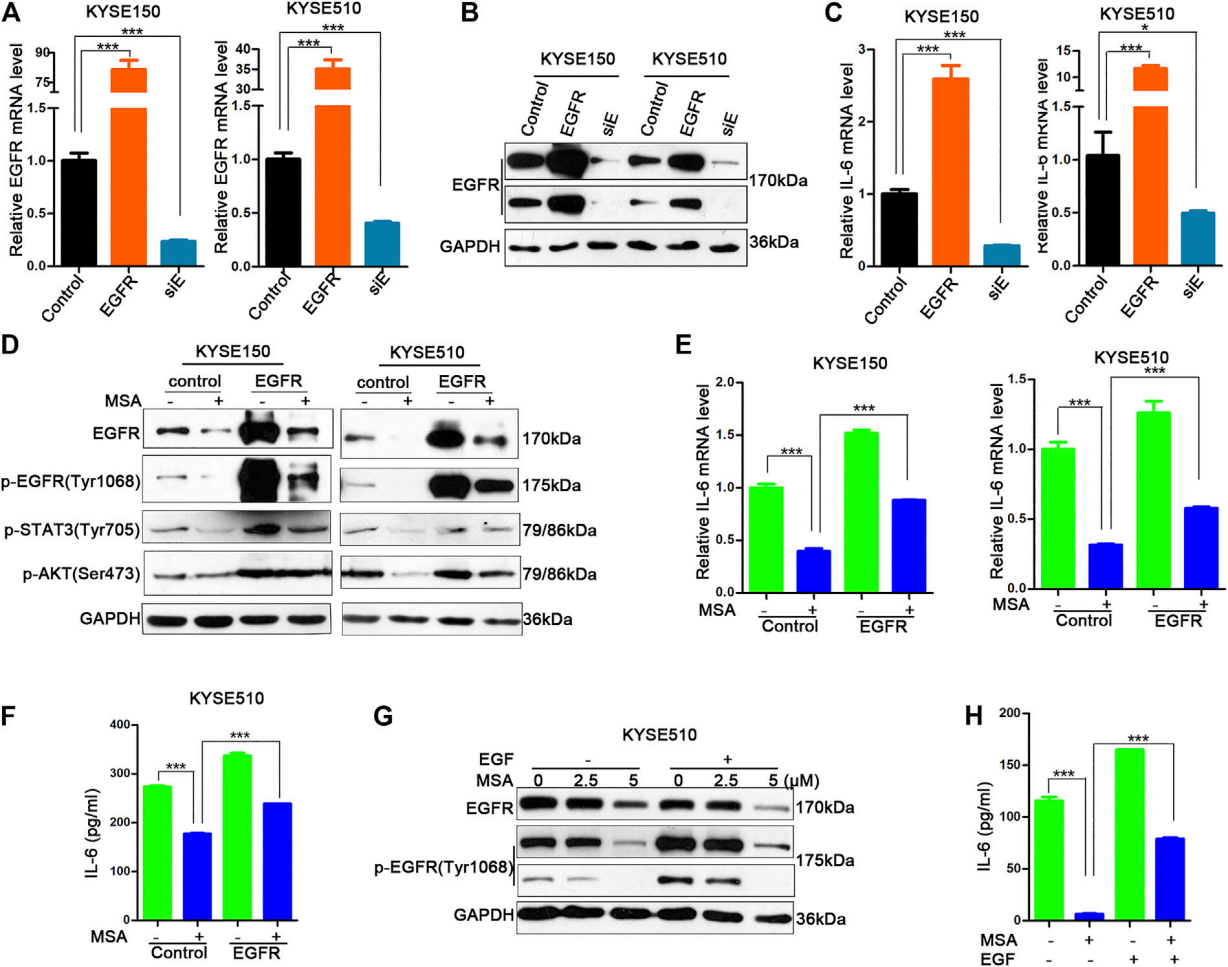
EGFR involved in MSA-induced IL-6 downregulation. **(A–C)** KYSE150 and KYSE510 cells were transfected with pcDNA6 (control), pcDNA6-EGFR (EGFR), or EGFR siRNA (siE), respectively. The cells were harvested after 48 h and EGFR mRNA **(A)** and EGFR protein **(B)** were detected. IL-6 mRNA levels were also detected by Real-time PCR **(C)**. **(D–F)** KYSE150 and KYSE510 cells were transfected with the pcDNA6, or pcDNA6-EGFR, and then treated with or without MSA (2 μM for KYSE150, 5 μM for KYSE510), respectively. EGFR, *p*-EGFR, *p*-Akt, p-STAT3 were detected by western blotting. IL-6 mRNA was detected by real-time PCR **(E)** and IL-6 protein in the supernant of KYSE510 was deceted by ELISA **(F)**. **(G,H)** EGF (25 ng/ml) was added in the supernant of KYSE510 cells for 12 h, EGFR, *p*-EGFR (short and long exposure) were detected by western blotting **(G)**. IL-6 protein in the supernant was detected in the supernant of KYSE510 cells treated with or without MSA (5 μM) by ELISA **(H)**. Bars represent the mean ± SD (*n* = 3) for each treatment. ***p < 0.001. Control: pcDNA6; EGFR: pcDNA6-EGFR; siE: EGFR siRNA.

We next examined whether MSA-induced IL-6 downregulation by depending on EGFR-mediated signal transduction. After transfection with pcDNA6, pcDNA6-EGFR or EGFR siRNA for 48 h in KYSE150 and KYSE510 cells, EGFR mRNA and protein were detected. The results revealed that EGFR expression plasmid could significantly upregulate EGFR mRNA and protein levels, and EGFR siRNA could efficaciously downregulate EGFR mRNA and protein, compared to the control cells, respectively (Figures 5A,B). Overexpression of EGFR increased IL-6 mRNA expression in both KYSE150 and KYSE510 cells, *vice versa* (Figure 5C).

EGFR overexpression increased the phosphorylation of EGFR, Akt, and STAT3 signaling proteins and could partly restored the inhitory effect of MSA treatment, including the phosphorylation of EGFR, Akt, STAT3 (Figure 5D) and IL-6 mRNA levels in both KYSE150 and KYSE 510 cells (Figure 5E). We also detected the levels of IL-6 in the supernant of KYSE510 cells that overexpressed EGFR accompanied with MSA treatment or not, the result showed that EGFR overexpression indeed upregulated IL-6 secretion and partly abolished the inhibitory effect of MSA treatment (Figure 5F). In addition, when we used EGF stimulation to active the EGFR pathway (Figure 5G), IL-6 secretion was higher in EGF-stimulated KYSE510 cells than the control cells that did not receive the EGF stimulation both in the groups with or without MSA treatment, respectively (Figure 5H).

### IL-6 Deficiency Attenuated the Tumor Inhibitory Effect of MSA

**FIGURE 6:**
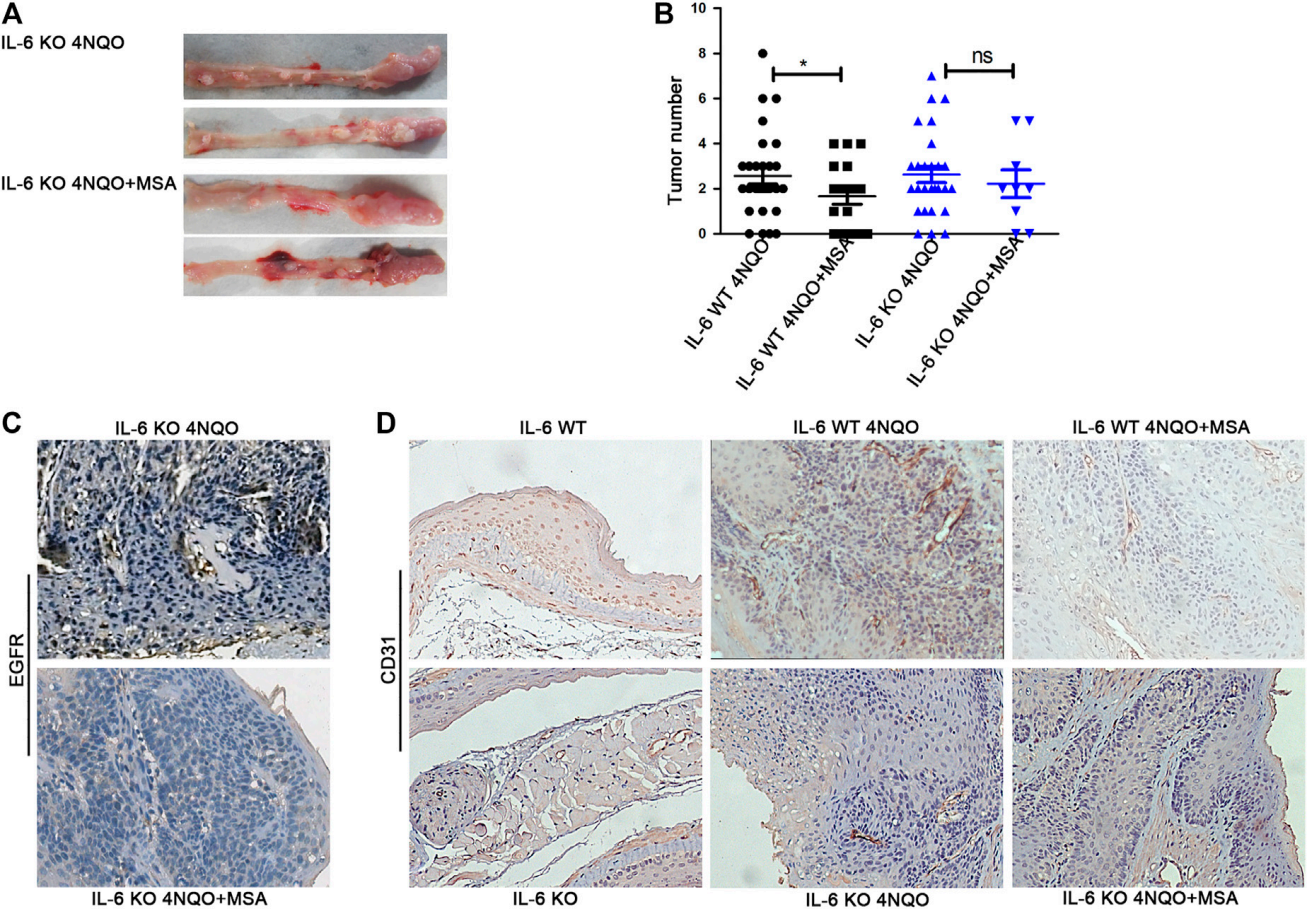
IL-6 deficiency attenuated the tumor inhibitory effect of MSA. **(A)** The esophageal tumors of IL-6^tm1Kopf^ (IL-6 KO) mice knock-out (KO) mice treated with 4NQO (100 μg/ml) in drinking water (4NQO) or the combination of 4NQO and MSA (4NQO + MSA) for 12 weeks. **(B)** Quantification of tumor numbers in 4NQO–treated mice with or without MSA treatment both in IL-6 Wildtype (WT) (normal C57) and IL-6 KO mice. All mice were with 4NQO exposure for 12 weeks, then mice were divided into the control or MSA treatment group. IL-6 WT 4NQO (*n* = 28), IL-6 WT 4NQO + MSA (*n* = 18), IL-6 KO-4NQO (*n* = 27), IL-6 KO 4NQO + MSA (*n* = 9). Bars represent the mean ± SD for each group. *p < 0.05. **(C)**Representative photomicrographs of immunostaining of EGFR in the esophageal tumor tissues of IL-6 KO mice treated with 4NQO or the combination of 4NQO and MSA (4NQO + MSA). **(D)** IHC staining of CD31 in normal esophageal tissues of IL-6 WT and KO mice, and the tumor tissues of 4NQO mice model treated with or without MSA. Magnification 100×.

To determine the role of IL-6 in tumorigenesis of esophageal cancer, we used IL-6 KO mice to generate esophageal cancer that induced by 4NQO. Our data showed that 4NQO-induced IL-6 KO mice formed almost the same tumors as 4NQO-induced IL-6 WT mice, indicating that lack of IL-6 may not affect tumorigenesis of esophageal cancer (Figure 6A). To further test the role of IL-6 in MSA-mediated inhibition of esophageal tumor growth, IL-6 KO mice were induced by 4NQO and then treated with MSA for 12 weeks. Interestingly, MSA treatment didn’t reduced the tumor number of 4NQO-induced IL-6 KO mice, MSA-mediated tumor suppression was abolished (Figures 6A,B). However, EGFR expression still could be down-regulated in 4NQO-induced IL-6 KO mice when treated with MSA (Figure 6C). To test whether MSA could inhibit EGFR-IL-6 axis and subsequently affect tumor angiogenesis, the tumor tissues were evaluated the expression of the endothelial staining marker CD31 by immunohistochemistry. As shown in Figure 6D, MSA treatment could obviously reduce vessel density and IL-6 deficiency could attenuate the effect. All these implicated that the anti-tumor effect of MSA was at least partly dependent on IL-6.

## Discussion

Selenium has been recognized as a chemotherapeutic agent in human beings for more than 100 years. One of the most exciting results demonstrated that intravenous injection of colloidal suspension of erythro-selenium beta resulted in significantly improvement in carcinoma of the alimentary tract (Watson-Williams, 1919). Previous studies showed that selenium treatment could downregulate EGFR mRNA levels in human biopsy-derived glima cells (Rooprai et al., 2007) and lung cancer cell lines (Shin et al., 2007). Sun *et al* reported Se-(methyl) selenocysteine hydrochloride (MSC) could inhibit pro-inflammatory responses by inducing miR-146a in *Staphylococcus* aureus-infected mouse mastitis model (Sun et al., 2017). miR-146a have been demonstrated to target EGFR directly (Li et al., 2010; Kogo et al., 2011). Consistent with these, we found MSA could up-regulate miR-146a at a dose- and time-dependent manner in ESCC cells; when we inhibited miR-146a with antagomir-146a, MSA-induced EGFR decrease was partly reverted, indicating that MSA-induced EGFR down-regualtion was dependent on miR-146a.

As a tyrosine kinase receptor, EGFR is capable to induce STAT3 phosphorylation (Levy and Darnell, 2002). STAT3 is also a transcriptional factor of IL-6. In the present study, we found MSA treatment could decrease the phosphorylation of STAT3, and the secretion of IL-6 in ESCC cells (Figure 4). Previous studies have already indicated the negative correlation between selenium and IL-6. For example, Zhou *et al* reported Selenium deficiency was present in Kashin–Beck disease (KBD), a chronic joint disease with chondral destruction, and IL-6 expression was elevated in the cartilages of KBD children (Zhou et al., 2014). Pei *et al* reported that sodium selenite pretreatment blocked the transcriptional factor NF-κB activation and inhibited IL-6 production in LPS-stimulated human PC3 cells (Pei et al., 2010). It has also been reported that MSA could inhibit tumor growth in colon cancer xenografts, and this inhibition was associated with a reduction of plasma tumor necrosis factor (TNFα)/IL-6 level (Zeng and Wu, 2015). Our findings showed that MSA treatment could inhibit IL-6 secretion, and EGFR was mediated MSA-induced IL-6 downregulation in ESCC, at least in part.

IL-6 is a cytokine that have extensive effects and its overexpression has been reported in almost all kinds of tumours (Kumari et al., 2016). Previous study showed that IL-6 is markedly associated with aggressive tumor behavior and poor outcomes in ESCC(Chen et al., 2013). The mice with IL-6 stimulation showed significantly increased myeloid derived suppressor cells (MDSCs) levels and an increased incidence of invasive esophageal tumor formation in the 4NQO-induced esophageal tumor animal model; however, blockade of IL-6 prevented induction of MDSCs and the incidence of 4NQO-induced invasive tumors (Chen et al., 2014). These data suggest that IL-6 blockade not only has direct intrinsic inhibitory effect on tumor cells, but also modulates the microenvironment toward an anti-tumor phenotype. In addition, previous study showed that selenium nanoparticles could increase the levels of cellular immunomodulatory components (granzyme B, IL-12, IFN-γ, and IL-2) to boost the immune response in mice bearing tumor exposed to crude antigens of 4T1 cells (Yazdi et al., 2015). Our experimental data showed that STAT3 activity in ESCC cells was markedly reduced when treated with MSA (Figures 4, 5), it is possible that the alteration also affects the antitumor immunity induced by MSA. All these provide hints that Se has potential to be applied to cancer treatment by modulating the immune microenvironment, but the underlying mechanism needs to be further clarified.

It is well documented that angiogenesis is necessary for the continued growth of tumors. IL-6, as a well-known angiogenic inducer, has been identified to enhance endothelial cell migration directly via IL-6R or through macrophage infiltration (Nilsson et al., 2005; Izumi-Nagai et al., 2007). Coward *et al* reported the therapeutic effect of siltuximab (anti-IL-6 antibody) was accompanied by reductions in angiogenesis in ovarian cancer cells xenograft models (Coward et al., 2011). Shinriki *et al* reported anti-Interleukin-6 receptor antibody suppressed tumor angiogenesis and *in vivo* growth of human oral squamous cell carcinoma (Shinriki et al., 2009). Here, we found MSA treatment could markedly decrease the density of tumor-associated vessels in tumors of 4NQO-induced wildtype mice. Although IL-6 deficiency did not affect esophageal tumorigenesis in mice (Figure 6), there are less vessels in the tumor tissue of 4NQO-induced IL-6 KO mice than that in 4NQO-induced wildtype mice. All these data suggest that MSA-induced IL-6 downregulation may impair tumor angiogenesis and then suppressed tumor growth *in vivo*.

In conclusion, we showed the intriguing possibility that MSA may have therapeutic value for the treatment of ESCC. MSA could downregulate EGFR via miR-146a and decrease IL-6 secretion, lead to substantial decrease in tumor angiogenesis to inhibit ESCC cell growth.

## Data Availability

The data generated in this article can be found in NCBI SRA using accession PRJNA742425.
