# Supplementary figures and images for: EGFR-IL-6 Signaling Axis Mediated the Inhibitory Effect of Methylseleninic Acid on Esophageal Squamous Cell Carcinoma
Source: Front Pharmacol. 2021 Jul 30;12:719785. doi: 10.3389/fphar.2021.719785 (PMC8363297; doi:10.3389/fphar.2021.719785)

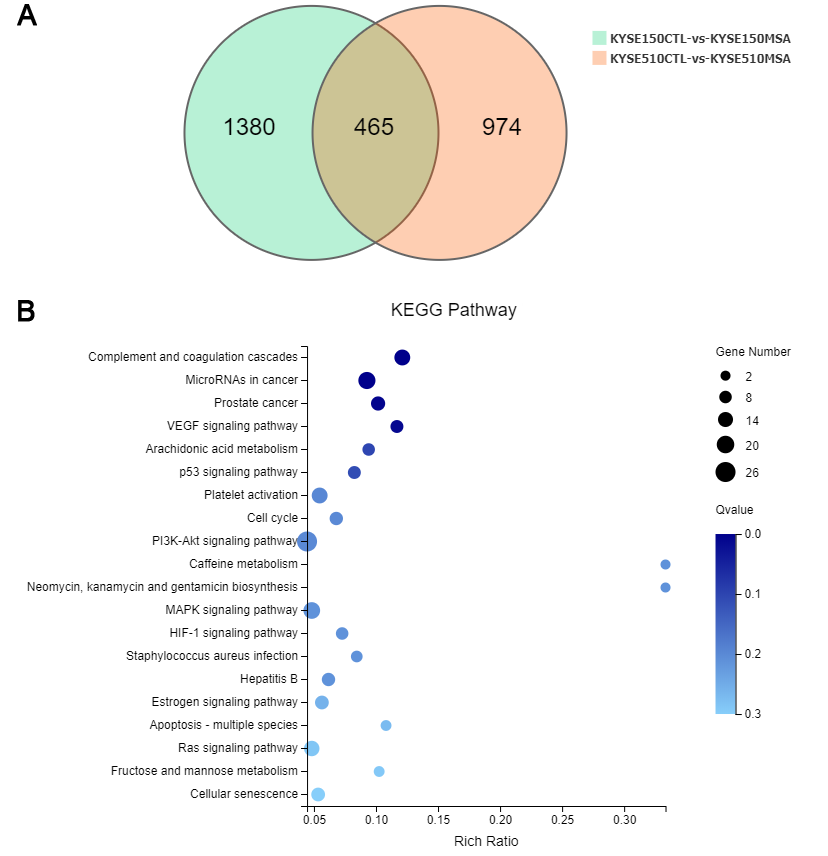

Supplement: Supplementary file 1 [file Image1.TIF]
